# Identification of key hub genes in pancreatic ductal adenocarcinoma: an integrative bioinformatics study

**DOI:** 10.3389/fbinf.2025.1536783

**Published:** 2025-03-28

**Authors:** Kankana Bhattacharjee, Avik Sengupta, Rahul Kumar, Aryya Ghosh

**Affiliations:** ^1^ Department of Chemistry, Ashoka University, Sonipat, Haryana, India; ^2^ Department of Biotechnology, Indian Institute of Technology Hyderabad, Kandi, Telangana, India

**Keywords:** PDAC, differentially expressed genes (DEGs), survival analysis, key hub-genes, gene-interaction network

## Abstract

Pancreatic Ductal Adenocarcinoma (PDAC) poses a significant health threat characterized by poor clinical outcomes, largely attributable to late detection, chemotherapy resistance, and the absence of tailored therapies. Despite progress in surgical, radiation, and chemotherapy treatments, 80% of PDAC patients do not benefit optimally from systemic therapy, often due to asymptomatic presentation or disease regression upon diagnosis. The disease’s progression is influenced by complex interactions involving immunological, genetic, and environmental factors, among others. However, the precise molecular mechanisms underlying PDAC remain incompletely understood. A major challenge in elucidating PDAC’s origins lies in deciphering the genetic variations governing its network. PDAC exhibits heterogeneity, manifesting diverse genetic compositions, cellular attributes, and behaviors across patients and within tumors. This diversity complicates diagnosis, treatment strategies, and prognostication. Identification of “Differentially Expressed Genes” (DEGs) between PDAC and healthy controls is vital for addressing these challenges. These DEGs serve as the foundation for constructing the PDAC protein interaction network, with their network properties being assessed for further insights. Our analysis revealed five key hub genes (KHGs): *EGF, SRC, SDC1, ICAM1 and CEACAM5*. The KHGs were predominantly enriched in pathways such as: ErbB signaling pathway, Rap1 signaling pathway, etc. Acknowledging the therapeutic promise and biomarker importance of PDAC KHGs, we have also pinpointed approved medications for the identified key genes. Nevertheless, it is crucial to conduct experimental validation on KHGs to confirm their effectiveness within the PDAC context. Overall, this study identified potential key hub genes implicated in the progression of PDAC, offering significant guidance for personalized clinical decision-making and molecular-targeted therapy for PDAC patients.

## Introduction

Pancreatic ductal adenocarcinoma (PDAC) is a complex and aggressive cancer in humans, ranking as the seventh most prevalent cause of cancer-related mortality. Its incidence is anticipated to elevate, potentially reaching the third position due to its rising occurrence and bleak prognosis (Kleeff et al., 2016). Despite advancements in surgical, radiation, and chemotherapy interventions, the majority of PDAC patients, approximately 80%, do not receive appropriate systemic therapy, largely because they either lack symptoms or experience disease regression upon diagnosis. (Ducreux et al., 2015). Consequently, the 5-year overall survival rate for PDAC remains significantly low at 3%–5%. However, less than 20% of all patients are eligible for surgery because most are diagnosed with either locally advanced or metastatic disease (Shen et al., 2018; Mar Kolbeinsson et al., 2023). Given the pressing necessity to deepen our comprehension of PDAC pathogenesis, there is an imperative demand for the identification of novel biomarkers (Sturm et al., 2022). These biomarkers could serve as potential prognostic indicators and uncover novel therapeutic targets, aiming to ameliorate the currently dismal treatment outcomes (Dayimu et al., 2023).

It is widely recognized that the abnormal activation or deactivation of genes significantly contributes to the development and advancement of cancer (Jones et al., 2008). Existing research on PDAC indicates that the abnormal expression of genes is a significant factor in the occurrence and advancement of this neoplasm (Tesfaye et al., 2019; Zhao et al., 2018). Over the past decades, studies have offered molecular insights into PDAC, highlighting specific genes such as KRAS, PI3K, PTEN, mTOR, and pathways related to apoptotic signals, cell cycle regulation, and cell adhesion (Zhang et al., 2023; Yinga et al., 2011; Sentia et al., 2016). Nevertheless, comprehending the pathological mechanism of PDAC poses significant challenges, leading to a dearth of efficacious medications and elevated medical expenses (O’Neill et al., 2012).

Various treatment modalities are employed in the management of pancreatic cancer, encompassing surgical resection, chemotherapy, adjuvant chemotherapy, targeted therapies, and immunotherapies tailored to specific targets. Adjuvant chemotherapy combines surgical resection, radiation, or targeted therapy with chemotherapy. FOLFIRINOX, an FDA-approved regimen for locally advanced and metastatic pancreatic cancer, consists of a combination of leucovorin calcium (folinic acid), fluorouracil, irinotecan, and oxaliplatin. Administered prior to surgical resection, FOLFIRINOX diminishes tumor size in patients with locally advanced disease stages, achieving overall response rates (ORRs) of less than 28% and an 11-month period without cancer progress (Fan et al., 2019; Faris et al., 2013). Targeted therapy focuses on various kinases, cancer-specific proteins, and receptors, including passive immunotherapy using monoclonal antibodies. Clinical trials are exploring drugs targeting EGFR, HER2, VEGF, MAPK, IGF-1R, c-Met, and PI3K/Akt/mTOR (Borja-Cacho et al., 2008).

Despite the array of therapies available, the survival rate for patients remains notably low, primarily due to late diagnosis of pancreatic cancer resulting from nonspecific symptoms and the limited efficacy of drugs (Trikudanathan et al., 2021). The paramount concern is early detection, achievable through the identification of Pancreatic cancer specific biomarkers and effective prognostic techniques. A pressing need exists for the development of anti-PaCa drugs with minimal side effects and precise cancer targeting (Roacho-Pérez et al., 2021). This research conducts a comprehensive transcriptome analysis of pancreatic cancer, utilizing both microarray and high-throughput sequencing data to acquire valuable insights into the molecular alterations taking place in cells throughout the course of disease advancement. The primary focus is on holistic gene expression profiling in pancreatic cancer. Additionally, this study seeks to provide insights into the exploration of PDAC key hub genes through diverse databases and bioinformatics approaches.

Previous meta-analyses in PDAC have primarily focused on integrating multiple microarray datasets to identify DEGs. For instance, a study by Li et al. analyzed 11 microarray datasets comprising 334 tumor samples and 151 non-tumor samples to identify gene signatures differentiating PDAC from normal pancreatic tissues (Liu et al., 2019). Another investigation by Zhang et al. combined two expression profiles, GSE16515 and GSE22780, to identify hub genes serving as potential biomarkers for PDAC diagnosis and therapy (Zhang et al., 2018). The novelty of the current study lies in the integration of both RNA-seq (GSE171485) and microarray (GSE71989 and GSE22780) data, providing a more comprehensive and up-to-date analysis. This approach not only validates previously identified DEGs but also uncovers novel gene expression patterns and potential therapeutic targets, thereby contributing to a deeper understanding of PDAC pathogenesis and treatment avenues. The integration of these datasets is crucial for several reasons. First, combining RNA-seq and microarray data leverages the strengths of both platforms, resulting in a more robust and comprehensive analysis. Second, the increased sample size enhances statistical power, allowing for the detection of subtle gene expression changes that may be missed in smaller studies. Third, the diversity in sample populations improves the generalizability of the findings, making them more applicable to a broader patient population. Finally, this integrative approach facilitates the identification of potential drug-gene interactions by providing a more complete picture of the molecular alterations in PDAC, thereby informing the development of targeted therapies.

Previous studies on PDAC have often been limited by smaller sample sizes or reliance on single-cohort analyses, potentially restricting the generalizability of their findings. For instance, earlier research identified highly expressed genes in PDAC but was constrained by the scope of data available at the time (aacrjournals.org). In contrast, the combination of GSE171485, GSE71989, and GSE22780 allows for a more extensive meta-analysis, leveraging a larger and more diverse sample pool. This approach not only validates previously reported DEGs but also uncovers novel gene expression patterns and potential therapeutic targets that may have been overlooked in earlier studies. The inclusion of both RNA-seq and microarray data further enriches the analysis, providing a more comprehensive understanding of PDAC’s molecular landscape and facilitating the identification of drug-gene interactions that could inform future treatment strategies.

It is interesting to be noted that, a disease typically arises from disruptions within the intricate web of interactions among related genes within cells, rather than solely from abnormalities in a single gene. This understanding has introduced a systemic approach to understand biological issues, emphasizing the importance of comprehending the collective impact of multiple genes and proteins on disease development and advancement. This approach underscores the significance of viewing living systems as interconnected networks. Hence, the concept of “Network Medicine” emerges, seeking to delve into the intricacies of diseases by systematically identifying their pathways and modules (Safari-Alighiarloo et al., 2014). Here, we have developed protein-interaction maps and analyzed these maps through network algorithms to understand the theoretical aspects of network maps.

The primary aim of this study is to enhance our comprehension of the genes or proteins implicated in the initiation and progression of PDAC disease, to facilitate the development of more efficacious treatment strategies.

## Material and methods

### Detailed description of the data sets used in the study

In our study, one RNA-seq dataset GSE171485, two microarray datasets GSE71989 and, GSE22780 were obtained from the NCBI GEO repository database (https://www.ncbi.nlm.nih.gov/geo/) utilizing two platforms (Affymetrix and Illumina) to analyze the human gene expression profiling between normal/healthy and pancreatic cancer patients. A detailed description of GSE datasets is mentioned (Supplementary Table S1). We omitted any samples subjected to drug treatment or associated with any other disease. The datasets GSE171485, GSE71989 and GSE22780 offer a comprehensive and diverse foundation for conducting a meta-analysis to identify differentially expressed genes (DEGs) and potential drug-gene interactions in PDAC. Each dataset contributes unique attributes in terms of data heterogeneity, sample size, population diversity and methodological approaches, enhancing the robustness and applicability of the findings.

### Data heterogeneity and methodological approaches

GSE171485 provides high throughput RNA-sequencing data from six PDAC specimens and six adjacent non-tumor tissues, offering deep insights into gene expression profiles with high sensitivity and specificity. In contrast, GSE71989 and GSE22780 utilize microarray platforms to analyze gene expression. GSE71989 includes data from 14 PDAC tissues and eight normal pancreatic tissues, while GSE22780 comprises profiling of eight matched tumor and adjacent normal samples. The combination of RNA-seq and microarray data introduce methodological heterogeneity that, when integrated, can mitigate platform-specific biases and provide a more comprehensive understaning of gene expression alterations in PDAC.

### Sample size and population diversity

The aggregated sample size across these datasets enhance the statistical power of the meta-analysis. GSE171485 contributes 12 samples, GSE71989 adds 22 and GSE22780 16, totaling 50 samples. The increased sample size allows for more reliable detection of DEGs and reduces the likelihood of false positives. Moreover, the inclusion of samples from different populations and institutions enhance the generalizability of the findings, ensuring that the identified DEGs are representative of diverse PDAC patient cohorts. Standardized preprocessing and batch effect correction ensure data comparability. By combining three datasets, a robust meta-analysis can be performed to identify reliable DEGs and explore drug-gene interactions in PDAC. The integration of these datasets enhance statistical power, cross validation and biological relevance, ultimately facilitating the discovery of potential therapeutic targets and drug repositioning strategies for PDAC treatment (Balasenthil et al., 2011; Jiang et al., 2016; Wu et al., 2021).

### Data analysis and retrieving genes with differential expression

We performed RNA-seq analysis on the dataset GSE171485. We downloaded the raw Fastq files having single-end data and checked the quality of the Fastq files with “FastQC” tool (v0.12.1) (Wingett and Andrews, 2018). Further, we proceeded with alignment with “STAR” (v2.7.10a) (Dobin et al., 2013) against “hg38” human reference genome (Guo et al., 2017) as reference. Then, we calculated the read counts using the “featureCounts” (subreads package v2.0.3) tool (Liao et al., 2014). Differential expression analysis was performed utilizing the “DESeq2” package in R (Love et al., 2014). To perform microarray analysis on the datasets, initially we normalized the datasets using “RMA” (McCall et al., 2010). To proceed with the microarray expression analyses, we used “Affy” package in R (Gautier et al., 2004). From these analyses, we retrieved the top upregulated and downregulated genes from both the datasets.

### Differential gene expression and network analysis

We used STRING v12.0 (SearchTool for the Retrieval of Interacting Genes/Proteins) database (https://string-db.org/) to build a protein-protein interaction (PPI) network of the common differentially expressed genes (DEGs) in humans (Damian et al., 2023). STRING can help to give information about either physical or functional associations of the protein-protein interaction map. These connections are sourced from text analysis of literature, co-expression examinations, genomics-contextual forecasts, computational projections, and high-throughput experimental findings, alongside the consolidation of existing insights from other databases. “Cytoscape” software (version 3.9.1) was used to visualize and analyze the protein-protein interaction map DEGs in our study (Paul et al., 2003).

### Functional annotation/gene ontology enrichment analysis

Functional analysis and Gene Ontology (GO) enrichment were performed using the DAVID Web server (https://david.ncifcrf.gov/). This web-based bioinformatics resource offers an accessible platform for researchers to comprehensively analyze differentially expressed genes, providing a suite of functional annotation tools. Utilizing DAVID tools, researchers can identify enriched biological themes, including Gene Ontology (GO) terms, discover functionally related gene groups, visualize genes on BioCarta and KEGG pathway maps, and explore many-genes-to-many-terms relationships in a 2D view. Additionally, the platform enables the search for functionally related genes not present in the original gene list, enhancing the understanding of the biological significance of the gene set under investigation (Da et al., 2009; Brad et al., 2022).

### Identification of key hub genes

Performing a comprehensive analysis is crucial to derive optimal insights from a specified biological network construction. The main goal in omics data analysis is to pinpoint pivotal hub genes, acting as molecular regulators. The process of identifying these crucial hub genes within the network of (DEGs) entails leveraging topological network attributes, particularly metrics such as degree, closeness centrality, and betweenness centrality (Bell et al., 1999). Nodes with high betweenness centrality, called bottlenecks, have been demonstrated to predict gene essentiality (Duron et al., 2009). These topological properties were computed using the Network Analyzer plug-in in Cytoscape-3.9.1 (Paul et al., 2003).

### Analyzing the drug-gene interaction

In order to find potential drugs for PDAC treatment, we employed the DGIdb (v4.2.0) web tool (Sharon et al., 2020), which is a repository of interactions between drugs and genes as well as genes that can be targeted by drugs.

### Survival analysis of the key hub genes

In order to understand disease biology and improve patient outcomes survival analysis of the key hub genes was performed using GEPIA (Gene Expression Profiling Interactive Analysis) (http://gepia.cancer-pku.cn/about.html) database (Tang et al., 2017).

## Results

### Quantification of the differentially expressed genes

**FIGURE 1:**
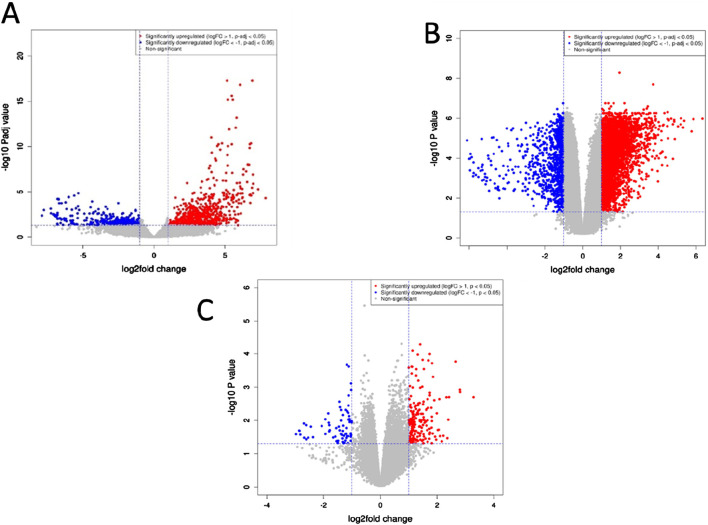
Volcano plots depicting genes exhibiting significant differences between pancreatic tumor tissues and neighboring non-tumor tissues across the three datasets. **(A)** GSE171485 **(B)** GSE71989 **(C)** GSE22780. The horizontal axis represents the fold-change (log scale), while the vertical axis represents the P-values (log scale). Each point represents a distinct gene, with red and blue denoting upregulated and downregulated genes, respectively.

To identify the DEGs we applied a cut-off of |log_2_foldchange|≥1 and padj (adjusted p-value) < 0.1. The padj value was computed using the Benjamini–Hochberg (BH) method to control for multiple testing. A total of 294 common DEGs were obtained from the three different datasets. Figure 1 presents volcano plots that illustrate the genes displaying significant differential expression between pancreatic tumor tissues and adjacent non-tumor tissues across the three datasets. These plots provide a visual representation of the statistical significance and magnitude of gene expression changes, with highly upregulated and downregulated genes distinctly highlighted. By integrating data from all three datasets, the volcano plots offer a comprehensive overview of key genes that may serve as potential biomarkers or therapeutic targets in pancreatic cancer research.

### Functional annotation of differentially expressed genes

**FIGURE 2:**
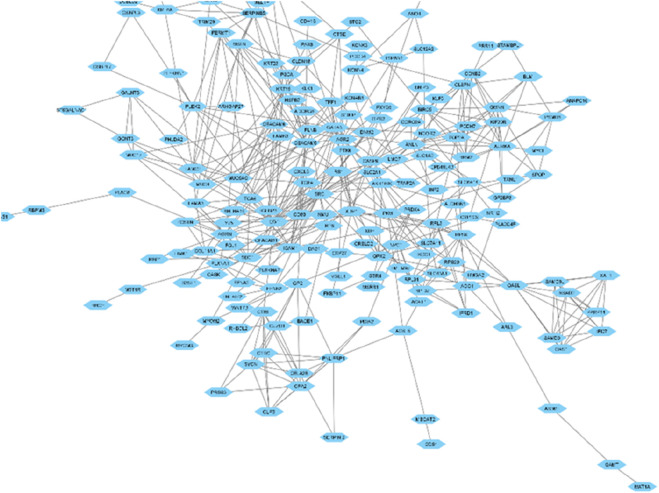
Network illustrating the interactions among proteins encoded by the genes that exhibit differential expression.

A total of 128 genes have been found to be upregulated and 90 genes have been found to be downregulated in our study. To understand the functions associated with the up and downregulated genes, Gene Ontology and KEGG pathway analyses of the common up and downregulated genes were performed using the DAVID server. The interaction network of the differentially expressed genes (DEGs) identified across the three datasets has been visually represented in Figure 2. This network illustrates the relationships and functional connections between the DEGs, providing insights into their potential roles in the underlying biological processes.

#### KEGG pathway analysis of upregulated DEGs

**FIGURE 3:**
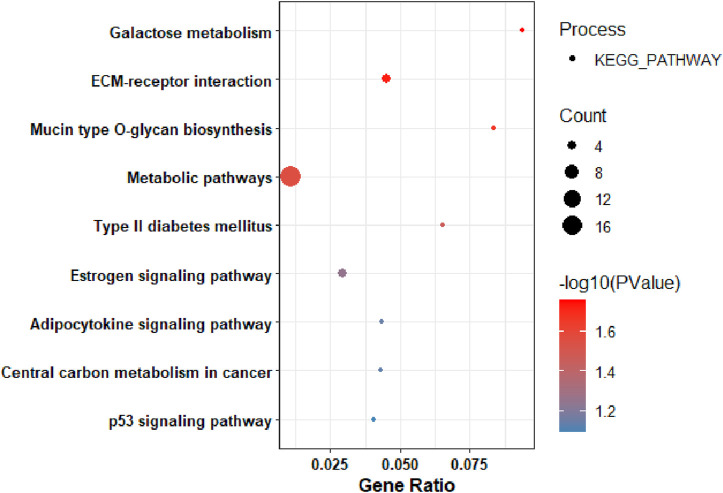
KEGG Pathway Analysis of among genes that were upregulated in pancreatic tumors compared to nearby non-tumor tissues.

Based on the KEGG pathway analysis, the upregulated DEGs were predominantly enriched in: galactose metabolism, ECM-receptor interaction, mucin-type O-glycan biosynthesis, metabolic pathways, type II diabetes mellitus (Figure 3). It is interesting to be noted that, significant gene count was associated with metabolic pathways.

#### Molecular Function, biological process and cellular component of upregulated DEGs

The Molecular Function (MF) of the upregulated DEGs was significantly enriched in: sterol transporter activity. The Biological process (BP) of the upregulated DEGs were mainly enriched in: cell adhesion, response to virus (Supplementary Figure S1). While the Cellular Component (CC) of the upregulated DEGs were mainly enriched in: plasma membrane and integral component of plasma membrane (Supplementary Figure S2).

#### KEGG pathway analysis of downregulated DEGs

**FIGURE 4:**
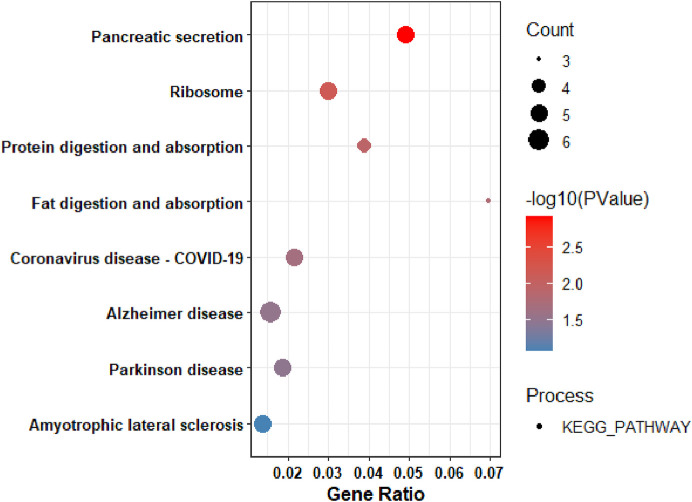
KEGG Pathway Analysis of the among genes that were downregulated in pancreatic tumors compared to nearby non-tumor tissues.

On the other hand, to gain insight into the functionalities of downregulated differentially expressed genes, Gene Ontology (GO) and KEGG pathway analyses were performed. Based on the KEGG pathway analysis, the downregulated DEGs were primarily enriched in: pancreatic secretion (Figure 4).

#### Molecular Function, biological process and Cellular Component of downregulated DEGs

The Molecular Function of the downregulated genes were significantly enriched in: hemoglobin alpha binding, organic acid binding, oxygen transporter activity. The Biological processes of the downregulated genes were: hydrogen peroxide catabolic process, cellular oxidant detoxification, cytoplasmic translation (Supplementary Figure S3). While, the Cellular Component (CC) of the downregulated DEGs were mainly enriched in: zymogen granule membrane, haptoglobin-hemoglobin complex, hemoglobin complex, endoplasmic reticulum, ribosome, cytosolic ribosome (Supplementary Figure S4).

### Discovery of the key hub genes through network analysis

To identify pivotal nodes within the network, centrality examines each node using metrics such as degree, betweenness and closeness. This approach was employed to pinpoint essential hub genes and bottleneck genes within scale-free biological networks based on their topological characteristics. Nodes with higher centrality values are instrumental in pinpointing biological entities that exert significant influence on the overall activities of the biological network. In order to enlist inferred genes in this network, we selected the top 20 genes based on degree, betweenness and closeness centralities, mentioned in Table 1. Additionally, by utilizing CytoHubba, extension of Cytoscape we identified the top 20 genes based on MNC (Maximum Neighbourhood Component) and EPC (Edge Percolated Component) properties, as outlined in Table 2. Afterwards, we aimed to identify shared genes that were present in a minimum of five attributes within the top twenty rankings across all assessed centralities and clustering techniques. These common genes were considered key hub genes. Notably, *EGF, SRC, ICAM1, CEACAM5,* and *SDC1* were frequently identified and assumed to be key hub genes.

**TABLE 1:** Genes Obtained from Differentially Expressed Gene interaction Networks.

S.N.	Degree	Betweenness centrality	Closeness centrality
1	*SRC*	*HBB*	*NKAP*
2	*EGF*	*ARL14*	*NKAPL*
3	*CEACAM5*	*SRC*	*P2RX1*
4	*ICAM1*	*EGF*	*SLC17A9*
5	*ANLN*	*PKM*	*SLC30A2*
6	*XBP1*	*CEACAM5*	*SLC39A8*
7	*AGR2*	*XBP1*	*HBB*
8	*KRT19*	*ANLN*	*ARL14*
9	*SDC1*	*AGR2*	*ALAS2*
10	*AURKA*	*GPX2*	*HBD*
11	*AGRN*	*OASL*	*TMEM92*
12	*PKM*	*RPS9*	*EPS8L3*
13	*MUC5AC*	*AGRN*	*SRC*
14	*ITGA6*	*ICAM1*	*EGF*
15	*BIRC5*	*SDC1*	*PKM*
16	*TOP2A*	*PLEK2*	*CEACAM5*
17	*OASL*	*GP2*	*ICAM1*
18	*GPX2*	*CTRL*	*CASP9*
19	*GMNN*	*KRT19*	*GATA3*
20	*GATA3*	*FLNB*	*SDC1*

**TABLE 2:** Genes Obtained from Differentially Expressed Gene Interaction Networks based on EPC and MNC Clustering.

S.N.	EPC	MNC
1	*EGF*	*EGF*
2	*SRC*	*SRC*
3	*ICAM1*	*CEACAM5*
4	*CEACAM5*	*ICAM1*
5	*ITGA6*	*ITGA6*
6	*SDC1*	*AURKA*
7	*KRT19*	*KRT19*
8	*MUC5AC*	*MUC5AC*
9	*AGRN*	*AGRN*
10	*XBP1*	*SDC1*
11	*GATA3*	*GMNN*
12	*PKM*	*ANLN*
13	*AGR2*	*TOP2A*
14	*ANLN*	*GATA3*
15	*MUC4*	*RRM2*
16	*TOP2A*	*RPS9*
17	*AURKA*	*MUC4*
18	*BIRC5*	*RPL3*
19	*CD80*	*KIF20B*
20	*TFF1*	*BIRC5*

EPC, edge percolated component; MNC, maximum neighbourhood component.

**FIGURE 5:**
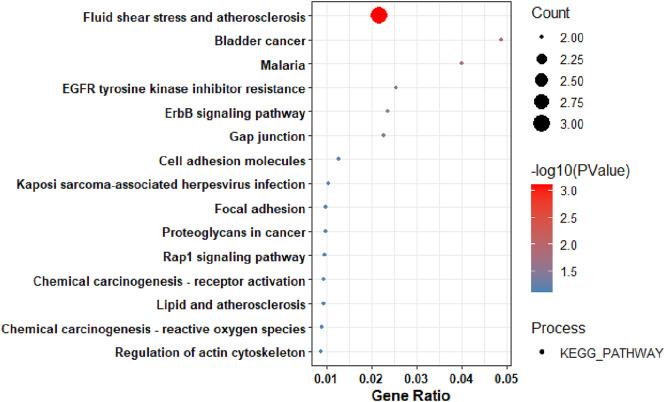
The KEGG Pathway analysis of the Key Hub-Genes.

The key hub genes showed enrichment in pathways linked to diverse processes such as fluid shear stress and atherosclerosis (Figure 5), with the significance of other pathways being less pronounced.

### Identification of drugs for the key hub genes

To understand the potential druggability of the identified key hub genes, approved drugs of the identified key hub-genes were discovered through DGIdb (https://www.dgidb.org/), a web-based database specializing in drug-gene interactions and druggable genes. The same has been described in Table 3.

**TABLE 3:** Identification of the approved drugs for the key hub genes.

S.N.	Key hub genes	Approved drugs
1	*EGF*	CETUXIMAB, PANITUMUMAB
2	*ICAM1*	LIFITEGRAST
3	*CEACAM5*	GEFITINIB
4	*SRC*	BOSUTINIB, DASATINIB, CERITINIB, PONATINIB, NINTEDANIB, LAPATINIB, CRIZOTINIB, TRAMETINIB, CLOZAPINE, GEMCITABINE, DOXORUBICIN, PACLITAXEL, VANEDETANIB, CISPLATIN
5	*SDC1*	Not available

### Survival analysis of the key hub genes

**FIGURE 6:**
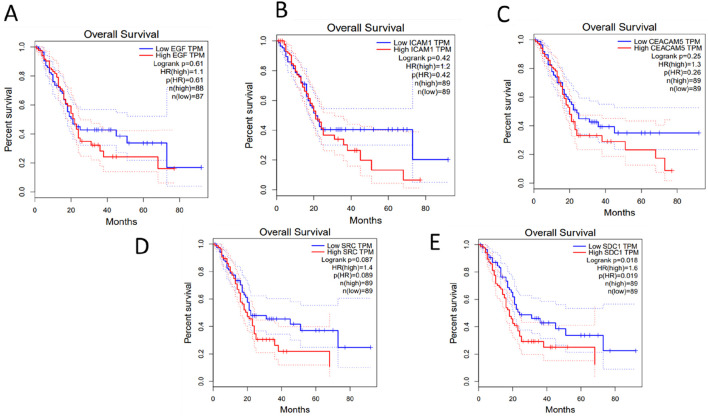
Survival analysis of the Key-Hub Genes **(A)** EGF, **(B)** ICAM1, **(C)** CEACAM5, **(D)** SRC, **(E)** SDC1.

It is important to be noted that, the survival outcomes of the genes can provide valuable prognostic information. By analyzing the expression levels of these genes in patient samples, researchers can predict the likelihood of disease progression, recurrence, or overall survival. GEPIA (Gene Expression Profiling Interactive Analysis) (http://gepia.cancer-pku.cn/about.html) database was used to perform survival analysis of the key hub genes. GEPIA provides a variety of customizable functions, such as comparing gene expression differences between tumor and normal tissues, profiling based on cancer types or stages, analyzing patient survival, identifying gene similarities, conducting correlation analysis, and performing dimensionality reduction analysis (Tang et al., 2017). It can be observed from Figure 6 that, high expression of the key hub genes significantly reducing the patients’ survival rate.

## Discussion

In this study, we analyzed gene expression profiles from three GEO datasets (GSE171485, GSE71989 and GSE22780) through comprehensive bioinformatics methods. Gene Ontology (GO) function and Kyoto Encyclopedia of Genes and Genomes (KEGG) pathway annotation of the overlapped DEGs discovered. Furthermore, by constructing a protein-protein interaction (PPI) network, we pinpointed several key hub genes closely associated with PDAC development and Survival analysis of the key hub genes performed to understand association between expression of the KHGs and PDAC survival rate.

As discussed earlier, *EGF, SRC, ICAM1, CEACAM5,* and *SDC1* were considered to be key hub genes in our study. In this study, *EGF* gene was found to be downregulated when PDAC was compared with healthy controls. The *EGF* (Epidermal Growth Factor) gene encodes a protein which is participated in cell growth, proliferation, and differentiation (Wee and Wang, 2017). Epidermal Growth Factor is a signaling molecule that plays a crucial role in the regulation of cell growth, development, and healing (Lindsey and Langhans, 2015). *EGF* binds to its receptor, the epidermal growth factor receptor (EGFR), triggering a series of intracellular signaling events that ultimately influence cell behaviour (Sabbah et al., 2020). This signaling pathway is important for normal physiological processes, such as tissue repair, as well as in the development of various organs and systems in the body (Berlanga-Acosta et al., 2009). Alterations or dysregulation of the *EGF* gene or its signaling pathway can be associated with various diseases, including cancers *viz.* colorectal cancer, non-small cell lung cancer (NSCLC), prostate and pancreatic cancers. Overexpression of *EGF* or mutations in its signaling pathway may lead to uncontrolled cell growth and contribute to the development and progression of cancer (Fenghua and Harris, 2014).

The nonreceptor tyrosine kinase c-Src (*SRC*), classified as a proto-oncogene, exhibits a correlation between its expression and activity with advanced malignancy and unfavorable prognoses in diverse human cancers. Originally recognized as the cellular counterpart of v-Src, the transforming gene product of the avian Rous sarcoma virus, *SRC* has been significantly implicated in the initiation, sustenance, advancement, and metastasis of various human cancers, including those affecting the prostate, lung, breast, colorectal and pancreatic tissues (Wheeler et al., 2009).

The *ICAM1* gene, which stands for Intercellular Adhesion Molecule 1, encodes a cell surface glycoprotein involved in immune responses and inflammation. *ICAM1* plays a crucial role in facilitating adhesion between cells, particularly between immune cells and endothelial cells. This adhesion is important for immune cell recruitment to sites of inflammation and infection. In the context of cancer, *ICAM-1* has been studied for its potential role in tumor progression and the immune response against cancer cells (Qiu et al., 2022).

The *CEACAM5* gene, also known as carcinoembryonic antigen-related cell adhesion molecule 5, is a gene that encodes a protein involved in cell adhesion and communication. This gene is a member of the carcinoembryonic antigen (CEA) family, which includes cell surface glycoproteins implicated in various physiological and pathological processes, including cancer. Overexpression of *CEACAM5* is related to numerous cancers *viz.* breast, colorectal and pancreatic cancers (Shi et al., 2022).

In this study, *SDC1* gene was found to be upregulated when PDAC was compared with healthy controls. The *SDC1* gene, also known as Syndecan-1, encodes a cell surface proteoglycan that is involved in cell adhesion, cell signaling, and the regulation of various cellular processes. Syndecan-1 belongs to the syndecan family of heparan sulfate proteoglycans. While it has essential roles in normal physiological processes, alterations in its expression and function have been associated with cancer *viz.* breast, multiple myeloma and pancreatic cancers (Sen et al., 2022).

The identification of hub genes such as EGF, SRC, SDC1, ICAM1 and CEACAM5 offer novel perspectives in PDAC research by highlighting previously underexplored molecular mechanisms and potential therapeucyic targets. While prior studies have identified various hub genes associated with PDAC, the focus on this specific set of genes provide unique insights into the disease’s pathogenesis. Previous bioinformatic analyses identified different sets of hub genes in PDAC. For example, Lu *et. al.* identified COL1A1, COL3A1 and FN1 as key genes involved in PDAC progression (Lu et al., 2018). Similarly, Dafrazi *et. al.* (Dafrazi et al., 2023) recognized COL1A1, COL3A1 and COL1A2 as significant in PDAC using comparable datasets. These studies primarily highlighted genes associated with the extracellular matrix and structural components of the tumor microenvironment. In contrast, the present study’s identification of EGF, SRC, SDC1, ICAM1 and CEACAM5 shifts the focus towards genes involved in cellular signaling, adhesion and immune interactions. EGF and SRC are integral to the EGFR signaling pathway, which is crucial for cell proliferation and survival. SDC1 (Syndecan-1) plays a role in cell-matrix interactions and has been implicated in tumor progression and metastasis. ICAN1 (Intracellular Adhesion Molecule 1) is involved in immune cell adhesion and transmigration, influencing tumor immune evasion. CEACAM5 (Carcinoembryonic Antigen-Related Cell Adhesion Molecule 5) is associated with cell adhesion and has been associated with tumor marker in various cancers. PDAC presents as a diverse condition, with a significant portion of patients being diagnosed at an advanced stage due to the lack of effective pre-detection measures. Despite extensive clinical and basic research efforts, there has been little notable improvement in the overall incidence and survival rates of PDAC over recent decades. It is recognized that, identifying key genes serving as diagnostic, prognostic or therapeutic biomarkers may vary depending on experimental conditions and other influencing factors. In this current investigation, bioinformatics analysis has been directed towards identifying key hub genes for PDAC.

The identified hub genes play crucial roles in the progression of PDAC and by being associated with differentially expressed genes (DEGs) that regulate metabolism and pancreatic secretion. These genes are involved in critical signaling pathways that drive PDAC tumorigenesis, including cell proliferation, adhesion, invasion and immune evasion. Their biological significance in PDAC is highlighted by their roles in key oncogenic pathways. The upregulated DEGs are mainly involved in metabolism, which is essential for supporting tumor growth and survival. Genes like EGF and SRC contribute to this metabolic shift by activating PI3K/AKT and MAPK signaling pathways, which enhance glucose uptake, lipid biosynthesis and amino acid metabolism-key hallmarks of cancer metabolism (Dosch et al., 2020). The downregulated DEGs in PDAC are mainly enriched in: pancreatic secretion pathways, suggesting a loss of normal pancreatic function. SDC1 (Syndecan-1) plays a role in maintaining pancreatic homeostasis by regulating cellular adhesion and signaling (Sanderson and Yang, 2008). Loss of pancreatic secretion-related genes along with alterations in ICAM1 and CEACAM5, contribute to the loss of normal exocrine function and promote tumor microenvironment remodeling. EGF and SRC are central to EGFR signaling, which drives PDAC cell proliferation, survival and resistance to apoptosis. Activation of Ras/Raf/MEK/ERK and PI3K/AKT/mTOR pathways by EGF promotes tumor growth, while SRC facilitates invasion and metastasis by enhancing cytoskeletal reorganization. SDC1 (Syndecan-1) modulates cell adhesion and interaction with the extracellular matrix (ECM), impacting tumor progression and chemotherapy resistance (Farhangnia et al., 2024). ICAM1, involved in inflammatory responses, contributes to immune evasion in PDAC by regulating leukocyte trafficking and tumor-associated inflammation. CEACAM5 (Carcinoembryonic Antigen-Related Cell Adhesion Molecule 5) is a marker of tumor progression and metastasis, implicated in cell adhesion and immune modulation (Qiu et. al., 2022; Shi et. al., 2022).

Activation of EGF and SRC supports oncogenic growth by enhancing cellular metabolism, proliferation, and evasion of apoptosis. SRC and SDC1 regulate ECM remodeling and integrin signaling, which contribute to PDAC cell migration and metastasis. ICAM1 and CEACAM5 are involved in immune escape mechanisms, helping PDAC evade host immune surveillance. Downregulation of genes involved in pancreatic secretion contributes to the destruction of normal pancreatic tissue, leading to the aggressive nature of PDAC. The interplay between upregulated metabolic genes and downregulated pancreatic secretion genes in PDC highlights a major shift towards tumor-driven metabolic adaptation and immune evasion. Hub genes like EGF, SRC, SDC1, ICAM1, and CEACAM5 serve as key oncogenic regulators, making them potential therapeutic targets. Targeting these pathways could disrupt PDAC progression, reduce metastasis, and enhance immune response, providing a strategic avenue for PDAC treatment.

The study relies on publicly available transcriptomic datasets, which may be limited in terms of sample size, clinical heterogeneity, or ethnic diversity. Integrating additional datasets, such as single-cell RNA sequencing or proteomic analyses, could improve the robustness of these findings. Future studies should validate findings across multiple PDAC subtypes, as different subgroups (e.g., classical, basal-like, immune-enriched) may exhibit varying gene expression patterns. While the study identifies key hub genes (KHGs) in PDAC, validating these genes requires rigorous experimental approaches. *In vitro* models, such as PDAC cell lines with gene knockdown or overexpression, and *in vivo* models, such as genetically engineered mouse models (GEMMs) or patient-derived xenografts (PDXs), could be utilized. Single-cell RNA sequencing and spatial transcriptomics could also refine our understanding of KHGs’ roles in different tumor microenvironments. CRISPR-based functional screens could systematically assess the necessity of these genes for tumor progression.

## Conclusion

We undertook an extensive examination utilizing one RNA-seq and two microarray gene expression datasets and compared PDAC with healthy pancreatic tissue. Our objective was to identify “Differentially Expressed Genes” (DEGs) and understand their biological insights through pathway enrichment analysis. Furthermore, we delved into the structural characteristics of the gene interaction network and obtained key hub genes. Furthermore, we identified drugs targeting these key hub genes. To comprehend the impact of both high and low expressions of the key hub genes linked to PDAC, we conducted survival analysis on these key hub genes. These genes are expected to have a pivotal influence on the advancement of PDAC.

## Details of the statistical packages and software tools used in this study

R version 4.1.0 used in this study.

Cytoscape 3.9.1 used for visualizing the protein interaction network data.

Cytoscape plugin “Cytohubba” used for predicting EPC, MNC properties.

Cytoscape plugin “Network Analyzer” used for network centrality measurements.

DAVID Web server (https://david.ncifcrf.gov/) used for GO- enrichment analysis.

STRING v12 database (https://string-db.org/) was used to construct a protein-protein interaction network.

DGIdb (https://www.dgidb.org/) web-based database of drug-gene interactions was used for drug identification for the identified key hub genes.

## Data Availability

The datasets presented in this study can be found in online repositories. The names of the repository/repositories and accession number(s) can be found in the article/Supplementary Material.
