# Supplementary material for: Identification of key hub genes in pancreatic ductal adenocarcinoma: an integrative bioinformatics study
Source: Front Bioinform. 2025 Mar 28;5:1536783. doi: 10.3389/fbinf.2025.1536783 (PMC11985535; doi:10.3389/fbinf.2025.1536783)
Supplement: Supplementary file 1 [file Table1.DOCX]

**Supplementary material**

**Identification of Key Hub-Genes in Pancreatic Ductal Adenocarcinoma: an Integrative Bioinformatics Study**

Kankana Bhattacharjee^1^ , Avik Sengupta ^2^, Rahul Kumar^2^, Aryya Ghosh^1^ *

^1^Ashoka University, Department of Chemistry, Sonipat, Haryana -131029

^2^ Department of Biotechnology, Indian Institute of Technology Hyderabad, Kandi, Telangana, 502284, India

**Table S1 : Description of GSE datasets used in this study**

| **S. No.** | **GSE Series ID** | **Samples** | **Platforms** | **Methods** | **Reference** |
| --- | --- | --- | --- | --- | --- |
|  | GSE171485 | 06 normal pancreas, 06 PDAC tissue | GPL11154 | Illumina | **[20]** |
| **2.** | GSE71989 | 08 normal**,** 13 PDAC tissue and 01 chronic pancreatitis tissue | GPL570 | Affymetrix | **[21]** |
| **3.** | GSE22780 | 08 normal, 08 tumor | GPL570 | Affymetrix | **[22]** |


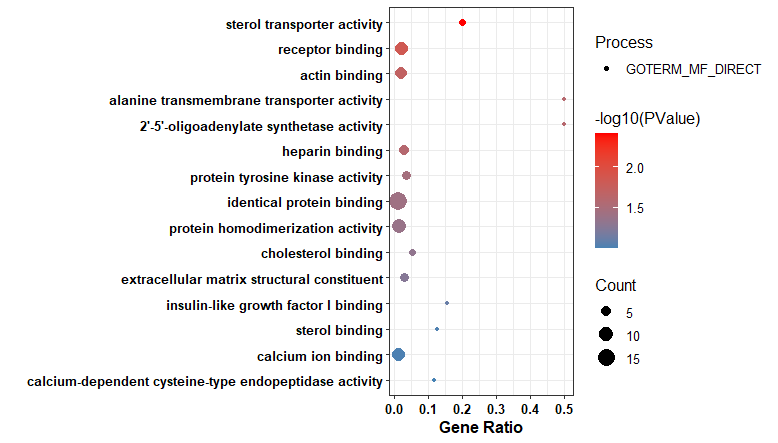


A


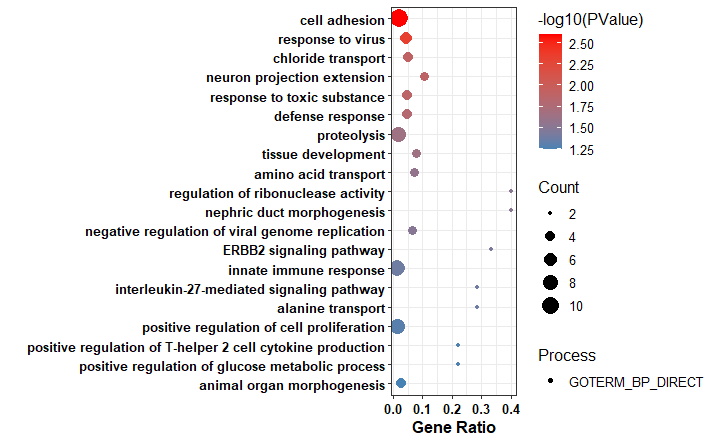


B

**Figure S1: Gene ontology terms among genes that were upregulated in pancreatic tumors compared to nearby non-tumor tissues. (A) Molecular Function, (B) Biological Process**


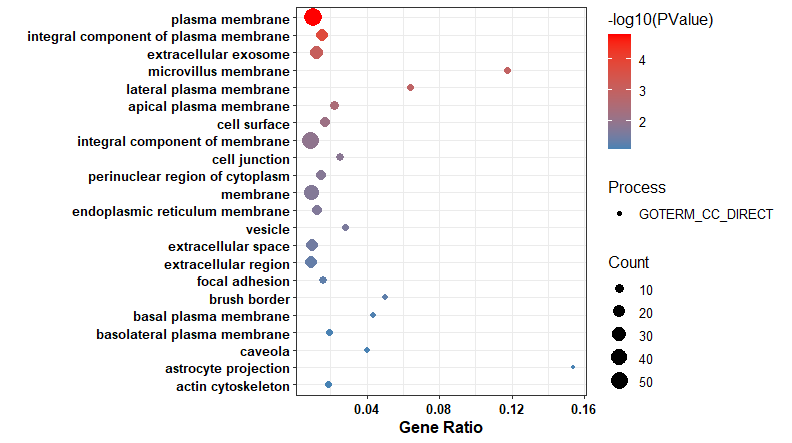


**Figure S2: Gene ontology terms (Cellular Component) among genes that were upregulated in pancreatic tumors compared to nearby non-tumor tissues**


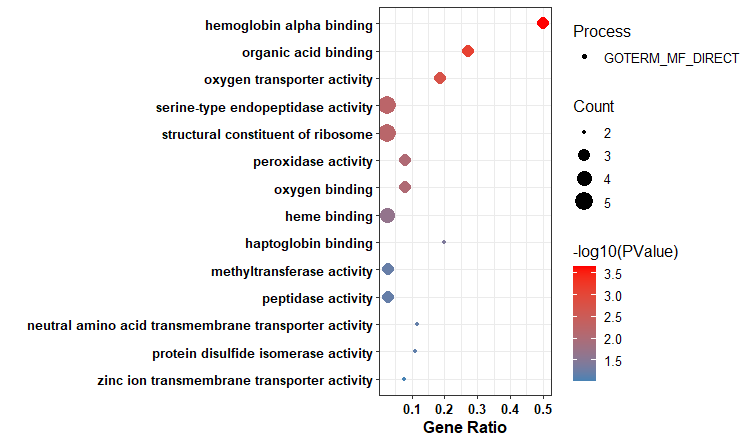

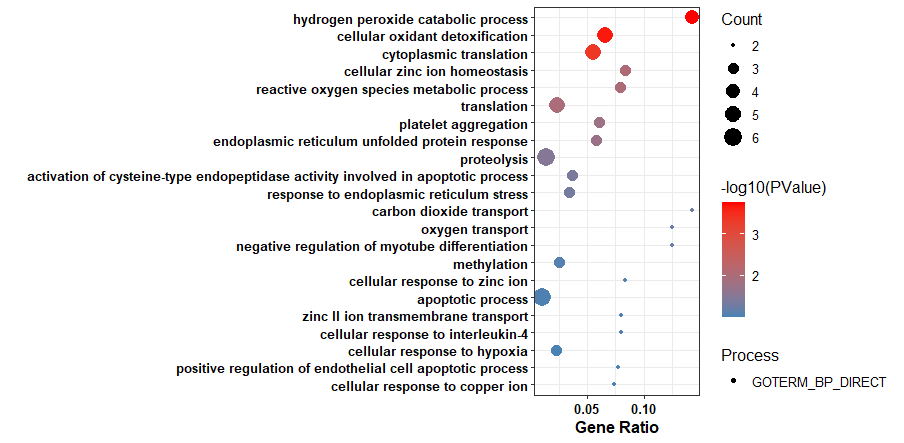


A

B

**Figure S3: Gene Ontology Analysis among genes that were downregulated in pancreatic tumors compared to nearby non-tumor tissues**. **(A) Molecular Function, (B) Biological Process**

**
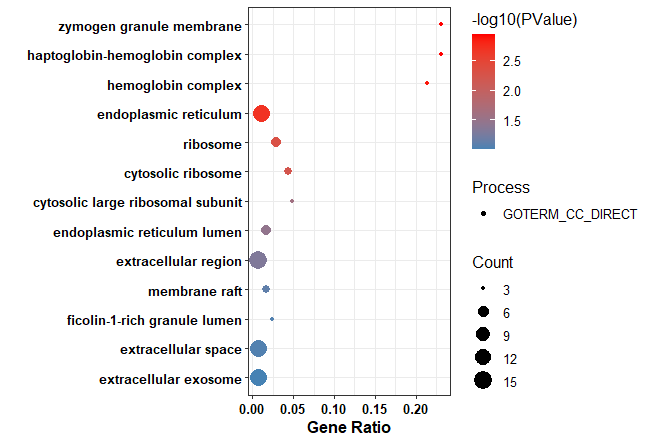
**

**Figure S4: Gene Ontology (Cellular Component) among genes that were downregulated in pancreatic tumors compared to nearby non-tumor tissues.**
